# Suppression of Rap1 Impairs Cardiac Myofibrils and Conduction System in Zebrafish

**DOI:** 10.1371/journal.pone.0050960

**Published:** 2012-11-30

**Authors:** Wei Dong, Zhenglin Yang, Fan Yang, Jialiang Wang, Yan Zhuang, Chongren Xu, Bo Zhang, Xiao-Li Tian, Dong Liu

**Affiliations:** 1 State Key Laboratory of Biomembrane and Membrane Biotechnology, Peking University, Beijing, China; 2 School of Life Sciences, Peking University, Beijing, China; 3 Institute of Molecular Medicine, Peking University, Beijing, China; Hong Kong University of Science and Technology, China

## Abstract

Numerous studies have revealed that Rap1 (Ras-proximate-1 or Ras-related protein 1), a small GTPase protein, plays a crucial role in mediating cAMP signaling in isolated cardiac tissues and cell lines. However, the involvement of Rap1 in the cardiac development *in vivo* is largely unknown. By injecting anti-sense morpholino oligonucleotides to knock down Rap1a and Rap1b in zebrafish embryos, and in combination with time-lapsed imaging, *in situ* hybridization, immunohistochemistry and transmission electron microscope techniques, we seek to understand the role of Rap1 in cardiac development and functions. At an optimized low dose of mixed *rap1a* and *rap1b* morpholino oligonucleotides, the heart developed essentially normally until cardiac contraction occurred. Morphant hearts showed the myocardium defect phenotypes, most likely due to disrupted myofibril assembly and alignment. *In vivo* heart electrocardiography revealed prolonged P-R interval and QRS duration, consistent with an adherens junction defect and reduced Connexons in cardiac myocytes of morphants. We conclude that a proper level of Rap1 is crucial for heart morphogenesis and function, and suggest that Rap1 and/or their downstream factor genes are potential candidates for genetic screening for human heart diseases.

## Introduction

In the heart, cardiomyocytes are responsible for pumping blood into the circulation, a process called the cardiac contraction, which is cAMP-dependent. An important cAMP sensor, Epac (exchange proteins directly activated by cAMP), has been extensively studied for its role and regulation in cardiovascular physiology and pathophysiology [1]–[3]. It is now clear that upon cAMP binding, the GEF (guanine-nucleotide-exchange factor) domain of Epac is exposed, allowing Epac to activate small Ras-like GTPase proteins, such as Rap1 (Ras-proximate-1 or Ras-related protein 1). In addition, Epac regulates the activity of various cellular compartments of cardiac myocytes and influences calcium homeostasis and excitation-contraction coupling, and thus, is potentially involved in many cardiovascular disorders, such as cardiac hypertrophy and remodeling [4], [5]. Recent experiments have demonstrated that Rap1 and its effectors mediate functions of Epac, perhaps by directly targeting or modulating the affinity of integrins at cell membranes [6], [7].

Rap1 is a small cytosolic protein that acts like a cellular switch for signal transductions. Rap1 carries an effecter domain similar to that of Ras, and functions to inhibit Ras signaling. As an evolutionarily conserved protein, Rap1 has been found in many animal species to play distinct roles in the worm (*C. elegans*), fly (*Drosophila*), zebrafish (*Denio rerio*), frog (*Xenopus*) and mouse. [8]–[13]. Previous studies have declared that Rap1 induces calcium release in cardiac excitation-contraction [14], a proposed function in cAMP metabolism and homeostasis in adrenergic signaling [15]. Originally thought to be a transformation suppressor with the ability to remodel the Ras-transformed phenotype, recent studies showed that Rap1 has many functions in cell attachment, migration and cell junction, and coupling of extracellular stimulation to intracellular signaling through second messengers. A vital role of Rap1 in the cardiovasculature was revealed, reflecting its multiple roles at different stages of heart development/remodeling and cardiac functions [16].

Rap1a knockout mouse are unexpectedly viable and fertile [17], presumably due to functional compensation by Rap1b. A study in frog and fish showed that a loss of both Rap1a and Rap1b functions can lead to severe convergence extension defect during gastrulation, and Rap1 serves as a component of the non-canonical Wnt pathway [18]. Moreover, in endothelial junction formation, knocking down *rap1b* alone causes specific cerebral hemorrhage [19], suggesting that Rap1a and Rap1b may be functionally redundant and yet possess individually unique functions. The fact that Rap1a and Rap1b have distinct roles in the cardiovasculature, from *in vitro* studies, emphasizes a critical role of Rap1 in heart functions however, its role in heart development remains blurry.

The coordinated cardiac conduction relies on gap junction (GJ) mediated electrical excitation [16], [20]. It is known that a connexon of each adjacent cardiomyocytes pairs to form a GJ, and a loss of the most abundant Connexin 43 (Cx43) leads to cardiac arrhythmias. On the other hand, cAMP-treated cardiac myocytes show increased Cx43 expression and the neoformation of functional GJs [21]. Interestingly, Somekawa et al showed that cAMP treatment increases GJs through activating Epac-Rap1 signaling and adherens junctions [22]; whereas the activation of Rap1 by Epac results in impeding ERK5, and thus in the decreasing of myocyte growth [23]. This suggests a role of Rap1 in myocardium function, thus likely to change cardiac development (the GJ remodeling).

We set to analyze if and how Rap1a and Rap1b regulate heart development and functions *in vivo*. The simplicity of the fish heart, with a single atrium and ventricle, and the ease of using time-lapsed recording tools can delineate complex functions of Rap1 during fish heart development, and distinguish its role in heart functions from developmental process. We carefully determined a combined dose of anti-sense morpholino oligonucleotides (MOs) against *rap1a* and *rap1b*, and produced specific heart, trunk and medial fin-fold defects. We found that loss of both Rap1a and Rap1b generates persistent myocardium defect phenotype as well as the first degree A-V block. At optimal MO injection, early heart development appeared to be normal until cardiac dilation progressively occurred from 20–24 hpf onward and some cardiac cells failed to move into the heart tube during heart morphogenesis. The 20 hpf is the stage when embryonic cardiac muscle cells gain their contractile/relaxation ability, and consuming/requiring more energy. Therefore, we believe that Rap1 plays an essential role in development/remodeling of cardiac functions, resembles the *in vivo* observation using mammalian cultured cardiac cells.

## Materials and Methods

### Zebrafish Maintenance and Transgenic Lines

Wild type Tubingen strain and all transgenic fish lines were raised under standard conditions [24]. *mp157e* is a transgenic line obtained from Tol2 mediated enhancer trap screening at the Peking University, named *Et(gata2a:EGFP)pku5*. *Tg(flk1:mCherry)pku6* is a home-made transgenic line using a *flk1:mCherry* plasmid (a gift from Shuo Lin’s lab at UCLA).

Ethics statement: All animal experiments were approved by Institutional Animal Care and Use Committee (IACUC) of Peking University. The reference from IACUC of Peking University is LSC-LiuD-01.

### RT-PCR and Gene Cloning

Total RNA was extracted from 50 wild type or morpholino injected embryos using the Trizon RNA isolation kit (Invitrogen) and used to synthesize first-strand cDNA by reverse transcriptase supplied with M-MLV kit (Promega). Specific primer sets for cDNA cloning of *rap1a* and *rap1b* were listed below. The long primers were used to synthesize mRNA in mis-expression experiments and the short primers for in situ anti-sense RNA probes.

*rap1a* Long sense: 5′- GCCCATCGATATGCGTGAATATAAGCTTGTG-3′

*rap1a* Long anti-sense: 5′- CCAACTCGAGTTACAGCAGGACACAGTTTGA -3′

*rap1a* Short sense: 5′-ATGCGTGAATATAAGCTTGTG-3′

*rap1a* Short anti-sense: 5′-TTACAGCAGGACACAGTTTGA-3′

*rap1b* Long sense: 5′- GGCCATCGATATGCGTGAATACAAGTTAGTAGTC-3′

*rap1b* Long anti-sense: 5′- ATTA CTCGAG TTAGAGCAACTGGCAGGTG-3′

*rap1b* Short sense: 5′- ATGCGTGAATACAAGTTAGTAGTC-3′

*rap1b* Short anti-sense: 5′-TTAGAGCAACTGGCAGGTG-3′

Primers set for RT-PCR of Cx43 and *rap1*.

Cx43 sense: 5′-TACTTGGGATTTGCTATGC-3′

Cx43 anti-sense: 5′-TCTCTGGAGCCTTTTCTTT-3′

*rap1a* sense: 5′- AAGCCGTGTTCATCATTC-3′

*rap1a* anti-sense: 5′- TAACCTTGCCACCCTAAA-3′

*rap1b* sense: 5′-CACAGCACAGTCCACCTT-3′

*rap1b* anti-sense: 5′-TGGGCAACAGTTCTTCAT-3′

### Morpholino Oligonucleotides and Microinjection

The antisense morpholino oligonucleotides against Rap1 genes, of which the sequences were previously published [18], [19] were purchased from Gene Tools, Inc (Philomath, Oregon).

*rap1a ATG MO: 5′ -TGGTGGCAGATTATTTCTTTTCACC-3′;*

*rap1b ATG MO: 5′-ACGCATTGTGCAGTGTGTCCGTTAA -3′;*

*rap1b splicing-blocking MO: 5′-CAATAGAAATGATGCAGAACTTGCC-3′;*

*Standard Control MO: 5′-CCTCTTACCTCAGTTACAATTTATA-3′;*

*p53 MO: 5′-GCGCCATTGCTTTGCAAGAATTG-3′;*

*rap1aATG MO (mis-match): 5′-TGcTcGCAcATTATaTgTTTTCACC-3′*

One-cell stage embryos were injected with 1 nl of various concentrations of individual (5 ng/nl) or mixed MOs (2.5 ng/nl each) dissolved in ddH_2_O from a Femtojet (Eppendorf). *rap1MO*s were co-injected with *p53* MO to prevent nonspecific cell death and/or artificial defect.

### mRNA Rescue Experiment

The cDNAs of *rap1a* and *rap1b* were cloned into pCS2+ and used for mRNA synthesis. Specific primer sets for *rap1a* and *rap1b* cloning are below.

*rap1a long S: (5′-GCCCATCGATATGCGTGAATATAAGCTTGTG-3′);*

*rap1a long As: (5′-CCAACTCGAGTTACAGCAGGACACAGTTTGA-3′);*

*rap1b long S: (5′-GGCCATCGATATGCGTGAATACAAGTTAGTAGTC-3′);*

*rap1b long As: (5′-ATTACTCGAGTTAGAGCAACTGGCAGGTG-3′);*

mRNA was synthesized *via* mMESSAGE mMACHINE SP6 system (Ambion), and purified with RNeasy Mini (QIAGEN).

Fertilized eggs were injected with 1 nl of mixed *rap1a*, *rap1b* and *p53* MOs and quickly were subjected to another injection of *rap1a* and *rap1b* mRNAs (1nl/egg). In control group, 1nl of ddH_2_O was used in the second injection.

### Whole-Mount in situ Hybridization and Immunostaining

In situ probes were synthesized with a DIG RNA labeling Kit (Roche), and purified with RNeasy Mini Kit (QIAGEN). Prehybridization and hybridization were performed at 65°C for 4 hours to overnight. The embryos were washed in 66% formamide/2×SSCT at 65°C for 30 min, in 2×SSCT at 65°C for 15 min, and twice in 0.2×SSCT at 65°C for 30 min. Then the embryos were incubated in blocking solution with 1/4000 volume of anti-digoxigenin-AP Fabfragments (Roche) at 4°C overnight. Embryos were washed in coloration buffer at room temperature for 25 min three times. The signals were detected by using BCIP/NBT Color Development Substrate (Promega). The reaction was stopped by washing with PBS.

Whole-mount in situ hybridization for *cmlc*2, *vmhc*, *rap1a* and *rap1b* was performed as described above. Whole-mount immunofluorescence experiment was performed as described [25], using primary monoclonal antibodies against sarcomeric myosin heavy chain (MF20)/S46 and Connexin 43 (Cx43) of gap-junction component (ANT1-GJA1,Sigma). Briefly, we fixed the young fish, and performed whole-mount antibody staining procedure. We then isolated the heart tube from each 3 dpf fish and pressed it on a glass slide for observation.

### Histology and Transmission Electron Microscopy

Whole embryos and isolated hearts at 3 dpf were fixed with 2.5% glutaraldehyde and 2.0% paraformaldehyde and post fixed in 1% osmium tetroxide. After washing, they were embedded in the Spur resin, and sectioned. The sections were stained with uranyl acetate and lead citrate and observed under a Philips CM100 transmission electron microscope at an accelerating of 100 KV.

### Imaging and Statistics

Embryos were examined with Zeiss Observer A1 and Leica S8APO, and photographed with a Zeiss Axiocam camera. 3-D images were obtained with confocal Zeiss LSM 510. Figures were processed with Adobe Photoshop CS4 software (Adobe).

**Figure 1:**
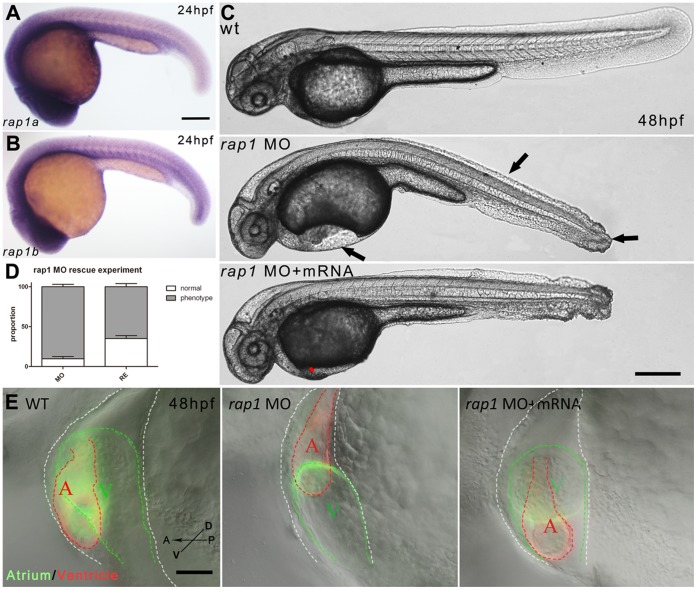
Rap1 knock-down led to abnormal heart and caudal fin development in zebrafish. Ubiquitous expressions of (A) *rap1a* and (B) *rap1b* were shown at 24 hpf. *rap1*MO injection led to the heart and caudal fin-fold phenotypes and curved trunk (arrows in C) at 48 hpf compared to wild type fish, and *rap1* mRNA partially rescued the heart malformation in *rap1* animals (C). The statistics indicates the proportion of fish embryos with heart phenotype was significantly decreased by *rap1* mRNA rescue (D). *mp157e* fish was used to show the heart morphology of wild type, morphant and rescued morphant at 48 hpf at a higher magnification, the atrium (red), ventricle (green) and cardiocoelom (white) are marked by color dotted lines (E). In *rap1*MO larvae, the heart apparently failed to develop properly, while *rap1* mRNAs partially restored the heart looping failure. Scale bar, 150 µm in A, B; 250 µm in C; 100 µm in E. Columns and error bars in D show mean±S.D. n>300 in each group.

To provide clear contrast of each part of the heart shown in Fig. 1E, we merged three photos of bright (DIC) and green fluorescent (highlighting the atrium) fields; and red field (the green fluorescence, highlighting the ventricle,was converted into red color via Photoshop).

TEM figures were further analyzed by counting numbers of thin and thick filaments in a 100 nm×100 nm of cardiomyocyte myofibrils region. From Cx43 antibody staining experiment, a 200 µm×200 µm of cardiomyocyte region (in confocol photo) was analyzed by ImageJ (National Institutes of Health) with manually set Cx43 signal threshold, and the result is represented as averaged value±S.D.

Statistic figures (Fig. 1D, 3D, and 3E) were generated by GraphPad Prism 5.01 (GraphPad Software, Inc.). Columns and error bars represented mean±S.D. Statistic symbols: ns for p>0.05; * for 0.01<p<0.05; ** for 0.001<p<0.01; *** for p<0.001.

### Electrocardiograph (ECG) Recording

The working platform was encased in a grounded metal cage. A pair of silver wires were suspended within pre-pulled borosilicate micropipettes (1.5 mm O.D., 2–3 MΩ in water; VitalSense Scientific Instruments Co., Ltd) filled with cell outside buffer (140 mM NaCl, 4 mM KCl, 1.8 mM CaCl_2_, 1 mM MgCl_2_, 10 mM Glucose, 10 mM Hepes, pH 7.4). Zebrafish embryos at 3 dpf were sedated in 0.63 mM tricaine (Ethyl 3-aminobenzoate methanesulfonic acid salt, 98%; Sigma) in E3 solution (containing 5 mM NaCl, 0.17 mM KCl, 0.33 mM CaCl_2_, and 0.33 mM MgSO_4_) and moved into M199 medium (Invitrogen) with 10% FBS and 20mM BDM (2, 3-butanedionemonoxime;Sigma). To exclude motion contamination, two micropipettes were used to disclose pericardial sac and expose heart to BDM. When the hearts stop beating, the positive and reference electrodes were introduced in atrium and ventricle respectively. The ECG signal was amplified (AM3000H-220-50, ADInstruments), digitized (ML870, ADInstruments), and recorded in 1-s sweeps with digital filtering at 0.5–10 Hz (Chart 5, ADInstruments).

## Results

### Loss of rap1 Functions Led to Specific Defects, Including the Heart Malformation, in Zebrafish

To investigate the role of *rap1* in zebrafish development, we used morpholino anti-sense (MO) oligonucleotides that were previously used to block the translational start site of *rap1a* [18] and a splice donor site of *rap1b* [19]. Because of the ubiquitous expression of both *rap1* genes at the embryonic stages (Fig. 1A-B), we reasoned that the dose of MO was crucial to distinguish artifacts from real loss-of-function phenotypes. During MO dose dependency tests, we observed variable phenotypes ranging from mild to severe. In low dose (<3 ng/nl of each) of morpholino injection, embryos and larvae survived normally. In high dose (>8 ng/nl of each), almost all injected embryos died or possessed severe abnormalities after 3dpf (data not shown). At 5 ng/nl, *rap1a*MO injection led to fairly specific heart, trunk muscle and caudal fin defects, while the intracranial hemorrhage phenotype was specific to *rap1b*MO injected animals (Table1, data not shown). Because the specificity of *rap1b*MO could easily confirmed by RT-PCR (data not shown; see also ref. 19), to ensure the specificity of *rap1a*MO, we used a mismatch *rap1a*MO to test if the consistent phenotypes observed in *rap1a* morphants was entirely due to *rap1a*MO. Similar to the uninjected control (182/185), the mismatch *rap1a*MO injection resulted in 118/122 normal fish at 3 dpf, indicating the specific loss of function phenotypes of *rap1a* morphants.

**Table 1:** Phenotype summary of the *rap1*MO fish.

MO oligo mix	Injected	Percentage of Embryos with Phenotype			
(5 ng/nl, each or mixed *rap1*MO)	(pooled from 3 experiments)	Heart	Somite	Tail	Intracranial hemorrhage(ICH)
***rap1a*** **+** ***p53***	683	85	89	78	3
***rap1b*** **+** ***p53***	540	31	36	10	67
***rap1a*** **+** ***rap1b*** **+** ***p53***	526	93	96	90	60

Because Rap1a and Rap1b may have redundant functions (Table1), to fully evaluate Rap1 functions and avoid obvious early developmental defects, we injected embryos with MO oligonucleotides each at 2.5 ng/nl, and observed constant defects, i.e., over 90% of double *rap1* knock-down morphants showed heart, trunk and caudal fin-fold phenotypes but intracranial hemorrhage remains a specific phenotype caused by *rap1b*MO (Fig. 1C, and Table1). This suggested additively specific effects of both morpholino oligonucleotides (Table1 and Fig. 1). Thus, in the following, we used *rap1*MO to refer to co-injection with MO oligonucleotides against both *rap1a* and *rap1b* each at 2.5 ng/nl. In *rap1*MO embryos, the heart was misshaped and the cardiocoelom expanded compared with those of wild-type fish (Fig. 1E). We also observed that *rap1MO* embryos had curved trunks, as well as blisters at the tip of the caudal fin (Fig. 1C, data not shown).

By injecting *rap1a* and *rap1b* mRNA into embryos pre-injected with *rap1*MO, we found that *rap1* mRNAs can rescue the heart deficiency in a dose dependent manner. There was no obvious rescue or side effect when small amount mRNAs was used (<0.1 ng/nl). While in a relatively low dose (0.25 ng/nl) of mRNAs, over 30% of *rap1*MO embryos had been partially rescued (Fig. 1D). Interestingly, when we further increased the dose of mRNA to 0.5 ng/nl, we observed no higher ratio of rescue but more distorted embryos (data not shown), suggesting that Rap1 level should be properly maintained *in vivo*.

**Figure 2:**
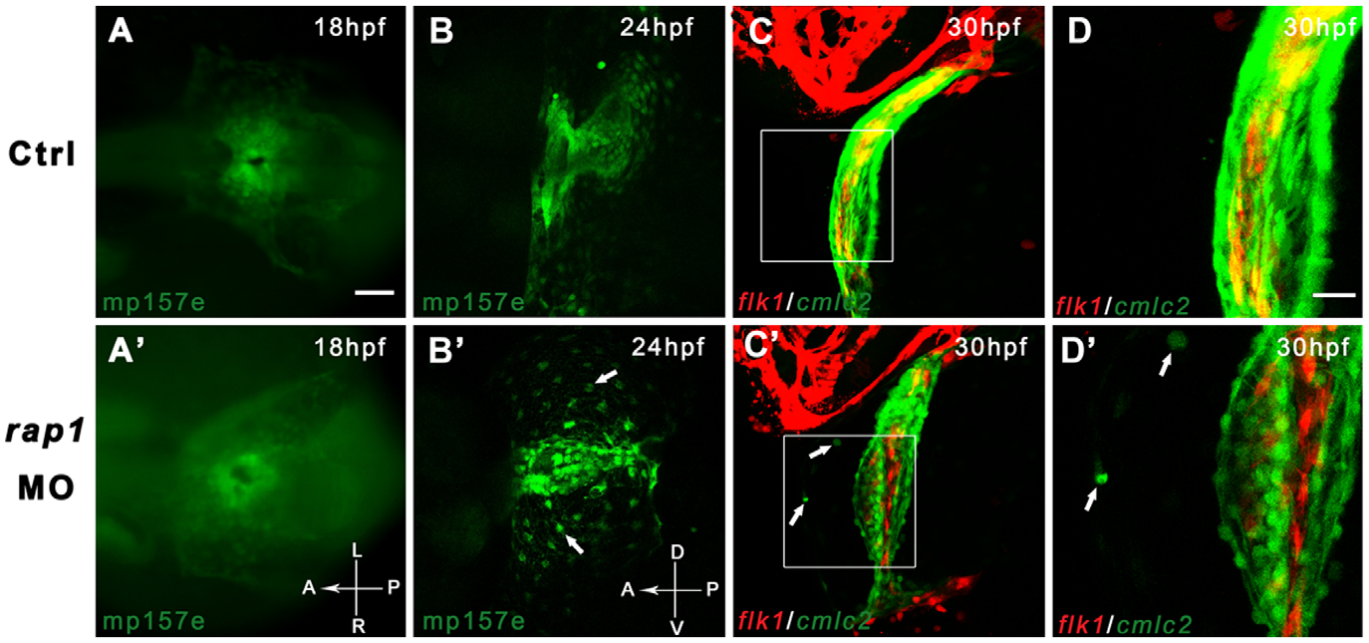
Time-lapsed observation revealed abnormal heart development in Rap1 knock-down zebrafish. Transgenic fish *mp157e* without any injection (A) and injected *rap1*MO (A') were indistinguishable in early heart development. By 24 hpf, *rap1*MO injected *mp157e* embryos, crossed in *flk1:mCherry* background, started to show abnormality in heart development and remodeling (B to B'), including the failure of heart tube extension, abnormal cardiac looping and incomplete heart chamber formation. In higher magnification views, the morphants show that a small portion of GFP labeled cardiac cells (arrows in B' to D') did not migrate properly to be eventually packed in the heart tube. Dorsal view, head to left in A to C and A' to C'. Lateral view, head to left in B to D and B' to D'. Scale bar, 100 µm in A to C, and A' to C'; 40 µm in D and D'.

Using transgenic embryos carrying cardiomyocyte-specific GFP (transgenic fish line *mp157e*, Fig. 2A–B and 2A’–B’), we observed malformed hearts in *rap1MO* embryos (Fig. 2). Although the initial heart development (18–22 hpf) was normal, *rap1MO* embryos showed cardiac defect from 24 hpf onward (Fig. 2B and 2B’), but *vmhc* probe still hardly distinguished the morphant heart from the control heart (Fig. S1C, C’) The characteristic phenotypes of *rap1MO* embryos included abnormal cardiac looping and incomplete heart chamber formation in transgenic *mp157e* carrying *flk1:mCherry* (Fig. 2C–D and 2C’–D’). However, nearby blood vessel cells expressing *mCherry* in *rap1MO* embryos appeared to be normal, indicating the specificity of cardiac defects caused by loss-of-Rap1 function. In older larvae (after 5dpf), *rap1MO* embryos showed persistent ventricular hypoplasia, heart dilation, and pericardial edema (data not shown), suggesting a correlation between *rap1* and cardiomyocyte function of the heart.

Early heart development, starting from around 10-somite stage, was essentially normal, judged by normal cardiac expression of *nkx2.5, gata4* and *tbx5* in *rap1MO* embryos until 21 hpf (Fig. S1, data not shown) when the cardiac myocytes started to contract. *rap1* morphants mainly had smaller and shorter heart tubes (68%, n = 81). At 48 hpf, some *rap1MO* embryos failed to form their heart ‘S’ loops (46%, n = 144), and the defect could be captured from confocal images of both ventral and lateral views (Fig. S2). Expression of *cmlc* and *vmhc* indicated the essential normal shapes of morphant hearts (Fig. S3A, 3A’, 3D and 3D’). At 72 hpf, the ventricular defect was more obvious, showing the pericardium edema (48%, n = 25) (Fig. S3B, B’). These results collectively indicated that *rap1* persistently and predominantly functioned to regulate ventricular development of the heart.

### rap1MO Animals had Disrupted Cardiac Structures

Starting from 24 hpf, some GFP^+^ (cardiac) cells failed to strictly follow their destined paths to the developing heart or to incorporate into the cardiac tissue when Rap1 function was lost (Fig. 2B’–D’, white arrows) and cardiac myocytes of heart tubes appeared to be loosely packed in *rap1MO* embryos (Fig. 2C–D, C’-D’). This is suggestive of heart structure and/or function defects.

**Figure 3:**
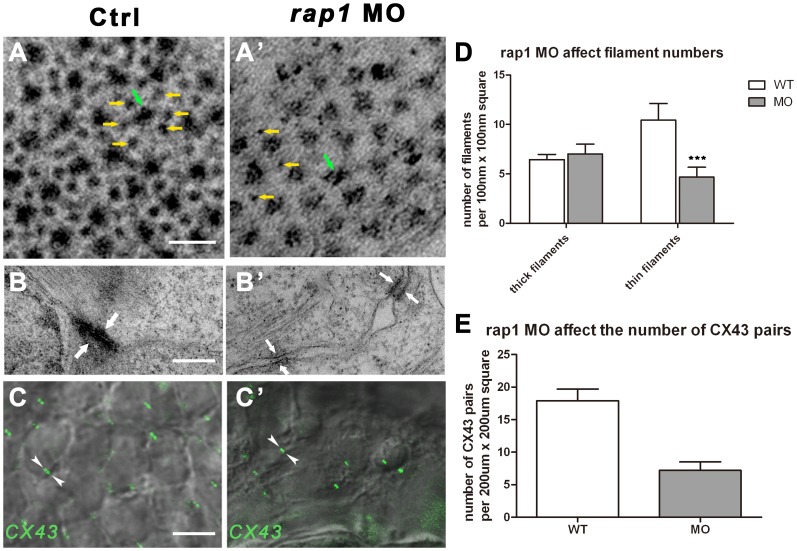
Ultra-structural changes of cell junctions and sarcomeres in Rap1 knock-down zebrafish heart. Transmission electronic scan revealed that the myofibril units, regularly organized into hexagonal lattices with thick (green arrow) and thin (yellow arrows) filaments (A) with adherens junctions (AJs) along the membrane of adjacent cardiac myocytes (B). In *rap1*MO heart, thick filaments (green arrow) number was not markablely changed but thin filaments (yellow arrows) number was significantly and statistically reduced (A' and D), and less myofibrils attached to AJs (arrows in B and B'), with lager space between the membrane of adjacent cardiac myocytes (B'). Antibody against Connexin 43 stained a significantly and statistically less GJ signal (arrowheads in C and C') in *rap1*MO cardiomyocytes at 3 dpf, compared to a normal heart (C, C', and E). Scale bar, 50 nm in A and A'; 350 nm in B and B'; 50 µm in C and C'. Columns and error bars in D and E showed mean±S.D. n = 9 in filaments experiment (D) for each group, and n = 10 in C×43 antibody staining experiment (E) for each group. Unpaired two-tailed t-test was used to test the significance between two columns in each group in D. *** in statistic graph represent p<0.001 in the t-test.

Compared to normal hearts, in which myofibrils were organized into hexagonal lattices with regularly arranged thick and thin filaments (Fig. 3A, 3A’), the units of myofibrils in *rap1MO* hearts had significantly fewer thin filaments but no difference in thick filaments compared to those of wild type fish heart (Fig. 3D). It was unclear if abnormal primary assembly of myofibrils was due to a lack of thin filaments; however, at least the absence of Z-discs may contribute to the formation of detached sarcomeric units (data not shown).

In normal heart, there were plenty of adherens junctions (AJs) along the membrane of adjacent cardiac myocytes; while in *rap1MO* hearts, there were only few or none AJs (data not shown). The close-up images of AJs in *rap1MO* hearts showed low electron density (Fig. 3B and 3B’) indicated that much less myofibrils were present, with obvious larger space between cell membranes of adjacent myocytes.

We then used antibody against C×43 to stain normal and morphant hearts at 3 dpf. The antibody highlighted 2 adjacent connexons that form GJ channels between two bordering cells (Fig. 3C, 3C'), and Cx43 was the predominant connexins in working cardiomyocytes. The number of connexon pairs, highlighted by the presence of Cx43 in ventricular cardiomyocytes, was significantly reduced in *rap1MO* hearts (Fig. 3E). Therefore, fewer GJs in ventricular cardiomyocytes, due to a suppression of *rap1*, may lead to heart dysfunction.

### Loss of rap1 Function led to Atrio-ventricular Block (A–V Block) in Zebrafish

Heart beating is the driving force to enable circulation of hematopoietic cells, and the blood flow promotes ventricular cells to enlarge and elongate in a process of heart development/remodeling. Upon imaging both control and morphant fish larvae in the cardiac regions, we found that in wild-type embryos, the heartbeats on average were 85/min at 36 hpf and 120/min at 72 hpf, while the averaged *rap1MO* heartbeats were 68 and 103, respectively. In addition to an obvious reduction in heart rate over time, ejection fraction and contraction volumes also decreased (data not shown).

**Figure 4:**
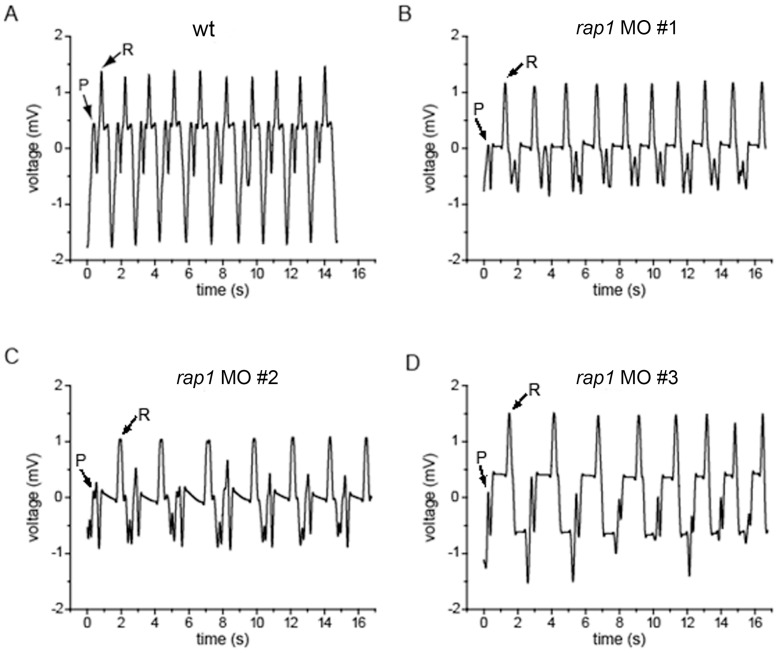
Rap1 knock-down zebrafish have prolonged P-R intervals, resembling the first-degree atrio-ventricular block. Zebrafish heart electrocardiography (ECG) was measured in wild type larvae (A), and *rap1*MO hearts at 3 dpf (B to D).

In addition, the GJ deficiency (Cx dependent) could be correlated to the conduction system of *rap1*MO animal hearts. To determine if there was any conduction problem, we measured the conduction of heart in 3dpf fish larvae in our own way, and in 10 control fish, we obtained fairly reproducible electrocardiography (ECG) readings. ECG of hearts at 3 dpf was recorded in sedated zebrafish with their hearts exposed to 20 mM BDM to avoid any motion interferences. The P and R waves were easily recognized by ECG recording of atrium or ventricular alone (data not shown). In control larvae, the P-R intervals were averaged at 0.664s (Fig. 4, A and Table 2); while in *rap1MO* fish (n = 12), the P-R intervals were averaged at 1.278s (Fig. 4, B–D and Table 2). The duration of QRS complex in *rap1MO* fish was also significantly longer than that of the wild type. Therefore, *rap1* deficiency in zebrafish hearts results in characteristic ECG changes such as first-degree atrio-ventricular block and the prolongation of the QRS duration, further suggesting that suppressing *rap1* may lead to abnormal heart function.

**Table 2:** Summary of ECG features.

ECG	WT	MO	p value
**Heart rate (beats/min)**	120±4	103±5	0.012
**P-R interval (s)**	0.664±0.047	1.278±0.142	0.000
**QRS duration (s)**	0.305±0.028	0.449±0.034	0.000
**Q-T interval (s)**	0.768±0.063	0.716±0.078	0.147

Note: Data was shown as mean ± S.D. P value was calculated by unpaired two-tailed t test.

## Discussion

In the present study, we provided *in vivo* evidence to reveal that Rap1 participates in cardiac development and function. We showed that by simultaneously knocking down two ubiquitously expressed Ras family members, namely zebrafish Rap1a and Rap1b, *rap1MO* larva exert abnormal cardiac AJs and show reproducible A-V block pattern and prolonged QRS duration, as well as slower heart beating activity and reduced ejection and contraction volumes (our unpublished observations). Comparison of ultra structures of normal and morphant hearts revealed that myofibril assembly and alignment were disrupted when *rap1* was suppressed, and the small number of thin filaments in cardiomyocytes may also contribute to slower heart beating rate observed in the morphants. These defects ultimately led to the heart failure.

It is known that the cardiac impulse conduction is in part governed by myocardium cell-cell coupling. GJs, in the ventricles of the heart and mainly composed of Cx43, enable low resistance communication between adjacent cells, thereby increasing the cell-cell coupling. Our finding that Cx43 level in the *rap1MO* heart was significantly lower than that of the normal heart (Fig. 3C, C’) is thus intriguing, because it is suggestive of a correlation between Rap1 and GJ, a fact that was previously demonstrated in cultured cells, in which the Epac-Rap1 signal accelerates AJ formation, leading to enhanced GJ formation [22]. Because previous studies also showed the Rap1 dependency of AJ positioning, and demonstrated that GJ assembly is dependent of and differentially regulated by AJ adhesion molecules [12], [26], the presence of poorly formed AJs in *rap1* morphant heart strongly suggests that the abnormal cell-cell coupling in cardiomyocytes, i.e., the A-V block and prolonged QRS duration in morphants, is ultimately due to an insufficient amount of Rap1. However, similar atrio-ventricular block measurements were evident in Cx40^−/−^ mouse hearts [27], rather than in Cx43^+/−^ mouse (Cx43*^−/−^* is embryonic lethal) [28]. Interestingly, a longer QRS duration was both evident in Cx40^−/−^ and Cx43^+/−^ hearts [27], [28], suggesting that Rap1 may target a Cx-complex composed of connexins(s) in addition to Cx43.

Numerous studies reveal that Rap1 is vital for effective cAMP signal transduction, which is downstream of Epac. However, whether Rap1 is involved in heart development is unknown. In previous studies, knocking down Rap1 functions either leads to severe defects in axial development (*rap1a*/*rap1b*MOs), making evaluation of heart development impossible, or the heart development is largely normal (*rap1b*MO). Because both *rap1a* and *rap1b* are ubiquitously expressed (Fig. 1A–B), it is expected that a complete lack of Rap1 during development is deleterious. In order to reveal the role of Rap1 in zebrafish heart development, we determined the dosage of *rap1* MOs, and found that Rap1 regulates overall development in a dose dependent manner. At a low level of anti-sense oligonucleotides, heart and somite defects were not observed by either *rap1a*MO or *rap1b*MO (2.5 ng/nl) alone, but only by their combination. The defects were certainly enhanced when both *rap1a* MO and *rap1b* MOs (each at 2.5 ng/nl) were used (Table 1). On the other hand, the fin-fold defect was specific to *rap1a*MO, while the intracranial hemorrhage was specific to *rap1b*MO, thus suggesting that the two zebrafish Rap1s are not entirely redundant in their functions.

In *rap1* morphants, the heart defect was first observable at 20–22 hpf, when initial cardiomyocyte contractions started during normal linear heart tube formation [29], and became more obvious when a small fraction of cardiac cells failed to integrate into the developing heart chambers (Fig. 2C–D, 2B’–D’). These observations led us to believe that the knock down of Rap1 function in zebrafish, at our experimental condition, has a limited effect on normal cardiac development at early stages (before 20 hpf). However, it was still surprising that massive heart failure eventually happened in the morphant. Because of a dramatic cardiac morphogenesis between 20 and 30 hpf, and a rash emergence of uncoordinated cardiac cells in the morphant hearts, it was reasonable to conclude that cardiomyocytes required Rap1 during heart remodeling process. It was possible that the lower than normal Rap1 level in morphant, initially compensated by maternally deposit Rap1 (maternal *rap1* mRNA is detectable at 1-cell stage, our unpublished data), or not required at all by the cardiomyocytes before cardiac contraction starts, may eventually not afford to mediate the rapid energy consumption in the developing heart. Rap1 signaling regulates cell junction integrity, and heart beating activity needs Rap1 during heart remodeling, thus upon the start of contractility and relaxation, low Rap1 leads to abnormal heart development and distorted cardiac structures in morphant. If this explanation is correct, one can predict that muscular contractions, starting at 19 hpf in the trunk myotomes, may lead to a similar developmental abnormality in trunk muscle. Indeed, with lower than normal Rap1 level, morphants showed mis-shaped somite boundaries and sarcomeres in the trunk, and their trunk muscle fibers are shorter (Fig. 1 and data not shown). It is reasonable to speculate that the trunk muscle fiber assembly and Z-disc are likely abnormal in *rap1* morphants.

Alternatively, the organ/tissue defects in *rap1*MO embryos/larvae may be due to distinct changes of tissue-specific Rap1 effectors/interacting factors in the heart, trunk and fin-fold. As a general GTPase, Rap1 activates its downstream targets/effectors to ultimately affect cell-cell interactions, and stands high in the hierarchy of cAMP dependent Epac-Rap1 regulatory pathway. It is possible that low Rap1 level fails to direct some downstream factors to reach theirs working threshold, thus leading to specific developmental defects. Therefore, one can predict that *rap1*MO animals resemble some, if not all, animals with inactivated or reduced individual Rap1 downstream factors. Zebrafish mutants, *mil*, *toh* and *ko157*, in which the sphingosine 1-phosphate (S1P) signaling is blocked and the cardiac precursors fail to move into primitive heart tube [30]–[32], are due to abnormal cell migration of cardiac precursors. It is known that Rap1 activation is important for S1P to direct cell trafficking and localization [33]. However, all three cardia bifida mutants are different from Rap1MOrphant in heart defects, indicating that Rap1 does not directly regulate S1P signaling in the heart. Strikingly, *rap1*MO animal shares an almost identical fin-fold defect with all three heart mutants, suggesting that the loss of Rap1 may inhibit S1P signaling directly during the fin-fold formation, although the nature of the fin-fold defect remains unknown. None of the studies using above cardia bifida mutants showed any trunk muscular defect(s), thus it is difficult to judge if they had trunk muscular defects, as in *rap1*MO animals. Nevertheless, it will be interesting to know which Rap1 downstream factor(s) can ensure heart functions and sub-structure development.

## Supporting Information

Figure S1 **The expression of** ***nkx2.5*** **in developing hearts.** The cardiac primordial marker gene,*nkx2.5*, was expressed essentially normal between 18–21 hpf (A-C), compared to that of wild type heart (D) Dorsal view, anterior to the left. Scale bar, 200 µm.
(TIFF)

Figure S2 **Morphant zebrafish has consistent defects in cardiac looping.** Compared to control *mp157e* embryos (A, B), *rap1*MO injected *mp157e* embryos, crossed in *flk1:mCherry* background, showed faulty heart tube ‘S’ loop and thin cavity, in both ventral and lateral views (C,D). Shown are ventral view (A, C) and lateral view (B, D), with anterior to the left.
(TIF)

Figure S3 **The cardiac looping defect was also evident by** ***in situ*** **hybridization.** At 24 hpf, both control and *rap1*MO animals showed alike *vmhc* expression pattern in their hearts. Unlike the wild type hearts (A, C, D), the heart tube failed to extend left (C, C’) at 24 hpf or failed to form ‘S’ loop (A, A’, D and D’) in *rap1*MO animals at 48 hpf. At 72 hpf, ventricular defects, including pericardium edema and abnormal chamber differentiation, was observed in *rap1*MO (B, B”). Ventral views were shown in A-A’, B-B’ and D-D’, anterior to the left;.dorsal view are shown in C-C’, anterior to the bottom. Scale bar, 150 µm in A, A', D and D'; 120 µm in B and B'; 80 µm in C and C'.
(TIF)

## References

[1] Laurent AC, Breckler M, Berthouze M, Lezoualc'h F (2012) Role of Epac in brain and heart. Biochem Soc Trans 40(1): 51–57. 22260665 10.1042/BST20110642

[2] Métrich M, Morel E, Berthouze M, Pereira L, Charron P, et al (2009) Functional characterization of the cAMP-binding proteins Epac in cardiac myocytes. Pharmacol Rep 61(1): 146–153. 19307703 10.1016/s1734-1140(09)70017-9

[3] Cazorla O, Lucas A, Poirier F, Lacampagne A, Lezoualc'h F (2009) The cAMP binding protein Epac regulates cardiac myofilament function. Proc Natl Acad Sci U S A 106(33): 14144–14149. 19666481 10.1073/pnas.0812536106 PMC2729034

[4] Hothi SS, Gurung IS, Heathcote JC, Zhang Y, Booth SW, et al (2008) Epac activation, altered calcium homeostasis and ventricular arrhythmogenesis in the murine heart. Pflugers Arch 457(2): 253–270. 18600344 10.1007/s00424-008-0508-3 PMC3714550

[5] Duquesnes N, Derangeon M, Métrich M, Lucas A, Mateo P, et al (2010) Epac stimulation induces rapid increases in connexin43 phosphorylation and function without preconditioning effect. Pflugers Arch 460(4): 731–741. 20585956 10.1007/s00424-010-0854-9

[6] Noda K, Zhang J, Fukuhara S, Kunimoto S, Yoshimura M, et al (2010) Vascular endothelial-cadherin stabilizes at cell-cell junctions by anchoring to circumferential actin bundles through alpha- and beta-catenins in cyclic AMP-Epac-Rap1 signal-activated endothelial cells. Mol Biol Cell 21(4): 584–596. 20032304 10.1091/mbc.E09-07-0580 PMC2820423

[7] Carmona G, Chavakis E, Koehl U, Zeiher AM, Dimmeler S (2008) Activation of Epac stimulates integrin-dependent homing of progenitor cells. Blood 111(5): 2640–2646. 18032709 10.1182/blood-2007-04-086231

[8] Bos JL, de Rooij J, Reedquist KA (2001) Rap1 signalling: adhering to new models. Nat Rev Mol Cell Biol 2(5): 369–377. 11331911 10.1038/35073073

[9] Bos JL, de Bruyn K, Enserink J, Kuiperij B, Rangarajan S, et al (2003) The role of Rap1 in integrin-mediated cell adhesion. Biochem Soc Trans 31(Pt 1): 83–86. 10.1042/bst0310083 12546659

[10] Rangarajan S, Enserink JM, Kuiperij HB, de Rooij J, Price LS, et al (2003) Cyclic AMP induces integrin-mediated cell adhesion through Epac and Rap1 upon stimulation of the beta 2-adrenergic receptor. J Cell Biol 160(4): 487–493. 12578910 10.1083/jcb.200209105 PMC2173739

[11] Price LS, Hajdo-Milasinovic A, Zhao J, Zwartkruis FJ, Collard JG, et al (2004) Rap1 regulates E-cadherin-mediated cell-cell adhesion. J Biol Chem 279(34): 35127–35132. 15166221 10.1074/jbc.M404917200

[12] Kooistra MR, Dubé N, Bos JL (2007) Rap1: a key regulator in cell-cell junction formation. J Cell Sci 120(Pt 1): 17–22. 10.1242/jcs.03306 17182900

[13] Pannekoek WJ, Kooistra MR, Zwartkruis FJ, Bos JL (2009) Cell-cell junction formation: the role of Rap1 and Rap1 guanine nucleotide exchange factors. Biochim Biophys Acta 1788(4): 790–796. 19159611 10.1016/j.bbamem.2008.12.010

[14] Oestreich EA, Malik S, Goonasekera SA, Blaxall BC, Kelley GG, et al (2009) Epac and phospholipase Cepsilon regulate Ca2+ release in the heart by activation of protein kinase Cepsilon and calcium-calmodulin kinase II. J Biol Chem 284(3): 1514–1522. 18957419 10.1074/jbc.M806994200 PMC2615515

[15] Oestreich EA, Wang H, Malik S, Kaproth-Joslin KA, Blaxall BC, et al (2007) Epac-mediated activation of phospholipase C(epsilon) plays a critical role in beta-adrenergic receptor-dependent enhancement of Ca2+ mobilization in cardiac myocytes. J Biol Chem 282(8): 5488–5495. 17178726 10.1074/jbc.M608495200

[16] Jeyaraj SC, Unger NT, Chotani MA (2011) Rap1 GTPases: an emerging role in the cardiovasculature. Life Sci 88(15–16): 645–652. 21295042 10.1016/j.lfs.2011.01.023 PMC3090149

[17] Duchniewicz M, Zemojtel T, Kolanczyk M, Grossmann S, Scheele JS, et al (2006) Rap1A-deficient T and B cells show impaired integrin-mediated cell adhesion. Mol Cell Biol 26(2): 643–653. 16382154 10.1128/MCB.26.2.643-653.2006 PMC1346907

[18] Tsai IC, Amack JD, Gao ZH, Band V, Yost HJ, et al (2007) A Wnt-CKIvarepsilon-Rap1 pathway regulates gastrulation by modulating SIPA1L1, a Rap GTPase activating protein. Dev Cell 12(3): 335–347. 17336901 10.1016/j.devcel.2007.02.009 PMC1857327

[19] Gore AV, Lampugnani MG, Dye L, Dejana E, Weinstein BM (2008) Combinatorial interaction between CCM pathway genes precipitates hemorrhagic stroke. Dis Model Mech 1(4–5): 275–281. 19093037 10.1242/dmm.000513 PMC2590810

[20] Raaijmakers JH, Bos JL (2009) Specificity in Ras and Rap signaling. J Biol Chem 284(17): 10995–10999. 19091745 10.1074/jbc.R800061200 PMC2670103

[21] Darrow BJ, Fast VG, Kléber AG, Beyer EC, Saffitz JE (1996) Functional and structural assessment of intercellular communication. Increased conduction velocity and enhanced connexin expression in dibutyryl cAMP-treated cultured cardiac myocytes. Circ Res 79(2): 174–183. 8755993 10.1161/01.res.79.2.174

[22] Somekawa S, Fukuhara S, Nakaoka Y, Fujita H, Saito Y, et al (2005) Enhanced functional gap junction neoformation by protein kinase A-dependent and Epac-dependent signals downstream of cAMP in cardiac myocytes. Circ Res 97(7): 655–662. 16123333 10.1161/01.RES.0000183880.49270.f9

[23] Dodge-Kafka KL, Soughayer J, Pare GC, Carlisle Michel JJ, Langeberg LK, et al (2005) The protein kinase A anchoring protein mAKAP coordinates two integrated cAMP effector pathways. Nature 437(7058): 574–578. 16177794 10.1038/nature03966 PMC1636584

[24] Westerfield M (2000) The zebrafish book. A guide for the laboratory use of zebrafish (Danio rerio). 4th ed. Eugene: Univ. of Oregon Press, 1.2–1.22.

[25] Alexander J, Stainier DY, Yelon D (1998) Screening mosaic F1 females for mutations affecting zebrafish heart induction and patterning. Dev Genet 22(3): 288–299. 9621435 10.1002/(SICI)1520-6408(1998)22:3<288::AID-DVG10>3.0.CO;2-2

[26] Knox AL, Brown NH (2002) Rap1 GTPase regulation of adherens junction positioning and cell adhesion. Science 295(5558): 1285–1288. 11847339 10.1126/science.1067549

[27] Hagendorff A, Kirchhoff S, Krüger O, Eckhardt D, Plum A, et al (2001) Electrophysiological characterization of connexin 40 deficient hearts–in vivo studies in mice. Z Kardiol 90(12): 898–905. 11826831 10.1007/s003920170060

[28] Guerrero PA, Schuessler RB, Davis LM, Beyer EC, Johnson CM, et al (1997) Slow ventricular conduction in mice heterozygous for a connexin43 null mutation. J Clin Invest 99(8): 1991–1998. 9109444 10.1172/JCI119367 PMC508024

[29] Bakkers J (2011) Zebrafish as a model to study cardiac development and human cardiac disease. Cardiovasc Res 91(2): 279–288. 21602174 10.1093/cvr/cvr098 PMC3125074

[30] Matsui T, Raya A, Callol-Massot C, Kawakami Y, Oishi I, et al (2007) miles-apart-Mediated regulation of cell-fibronectin interaction and myocardial migration in zebrafish. Nat Clin Pract Cardiovasc Med 4 Suppl 1: S77–82. 17230219 10.1038/ncpcardio0764

[31] Osborne N, Brand-Arzamendi K, Ober EA, Jin SW, Verkade H, et al (2008) The spinster homolog, two of hearts, is required for sphingosine 1-phosphate signaling in zebrafish. Curr Biol 18(23): 1882–1888. 19062281 10.1016/j.cub.2008.10.061 PMC2741689

[32] Kawahara A, Nishi T, Hisano Y, Fukui H, Yamaguchi A, et al (2009) The sphingolipid transporter spns2 functions in migration of zebrafish myocardial precursors. Science 323(5913): 524–527. 19074308 10.1126/science.1167449

[33] Durand CA, Westendorf J, Tse KW, Gold MR (2006) The Rap GTPases mediate CXCL13- and sphingosine1-phosphate-induced chemotaxis, adhesion, and Pyk2 tyrosine phosphorylation in B lymphocytes. Eur J Immunol 36(8): 2235–2249. 16821235 10.1002/eji.200535004

