# Supplementary figures and images for: Suppression of Rap1 Impairs Cardiac Myofibrils and Conduction System in Zebrafish
Source: PLoS One. 2012 Nov 30;7(11):e50960. doi: 10.1371/journal.pone.0050960 (PMC3511394; doi:10.1371/journal.pone.0050960)

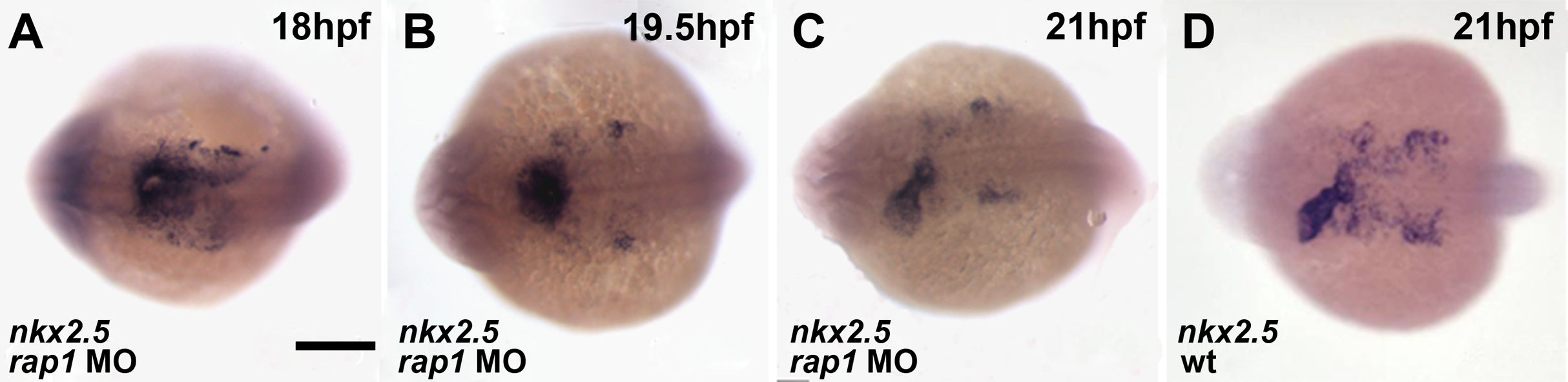

Supplement: Figure S1 — The expression of nkx2.5 in developing hearts. The cardiac primordial marker gene,nkx2.5, was expressed essentially normal between 18–21 hpf (A-C), compared to that of wild type heart (D) Dorsal view, anterior to the left. Scale bar, 200 µm. (TIFF) [file pone.0050960.s001.tif]

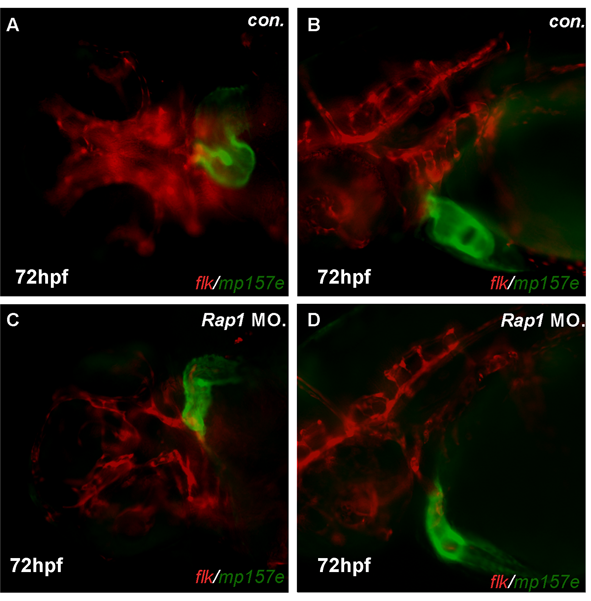

Supplement: Figure S2 — Morphant zebrafish has consistent defects in cardiac looping. Compared to control mp157e embryos (A, B), rap1MO injected mp157e embryos, crossed in flk1:mCherry background, showed faulty heart tube ‘S’ loop and thin cavity, in both ventral and lateral views (C,D). Shown are ventral view (A, C) and lateral view (B, D), with anterior to the left. (TIF) [file pone.0050960.s002.tif]

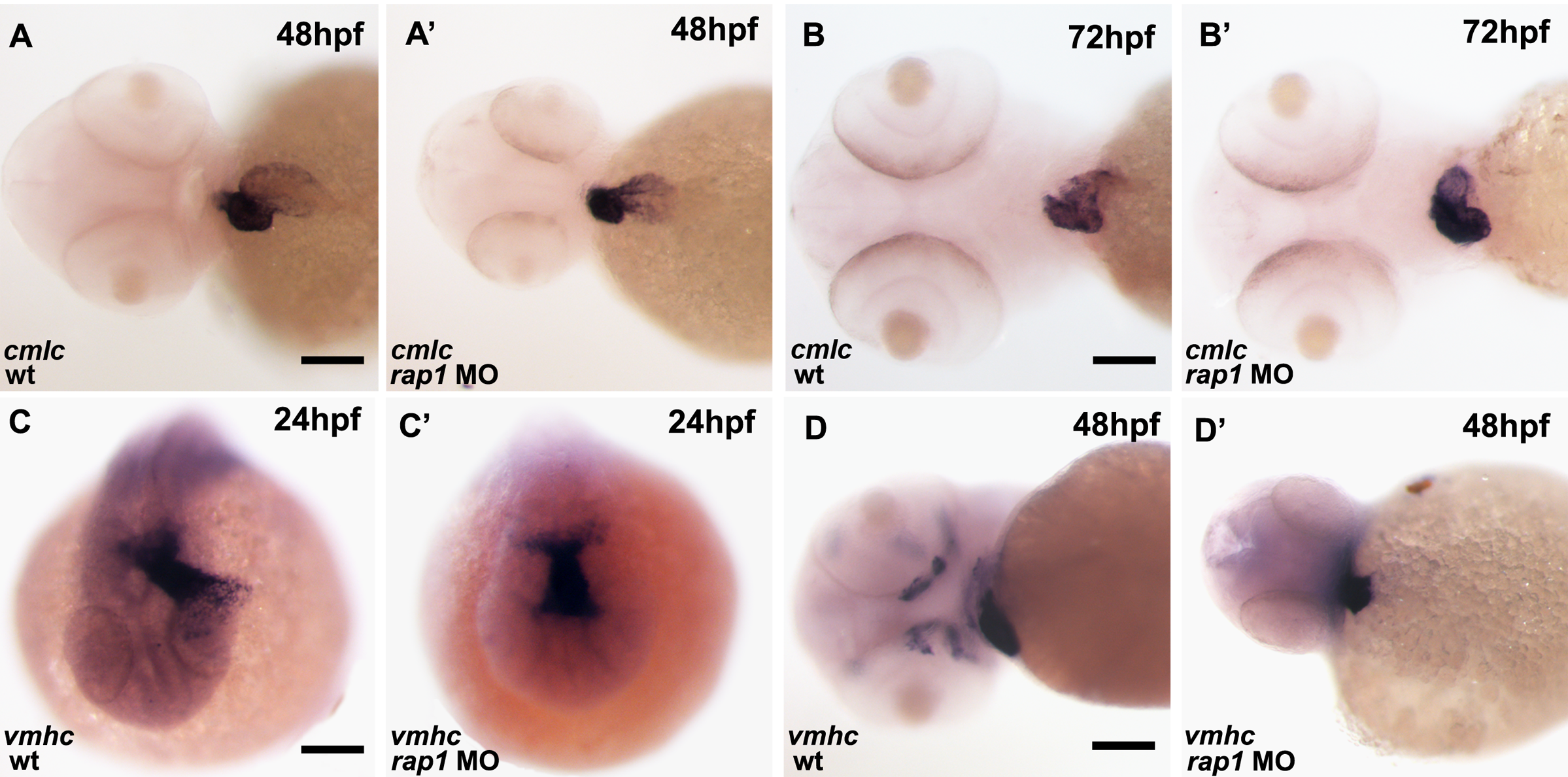

Supplement: Figure S3 — The cardiac looping defect was also evident by in situ hybridization. At 24 hpf, both control and rap1MO animals showed alike vmhc expression pattern in their hearts. Unlike the wild type hearts (A, C, D), the heart tube failed to extend left (C, C’) at 24 hpf or failed to form ‘S’ loop (A, A’, D and D’) in rap1MO animals at 48 hpf. At 72 hpf, ventricular defects, including pericardium edema and abnormal chamber differentiation, was observed in rap1MO (B, B”). Ventral views were shown in A-A’, B-B’ and D-D’, anterior to the left;.dorsal view are shown in C-C’, anterior to the bottom. Scale bar, 150 µm in A, A', D and D'; 120 µm in B and B'; 80 µm in C and C'. (TIF) [file pone.0050960.s003.tif]
